# To Be or Not To Be Vaccinated: That Is a Question in Myasthenia Gravis

**DOI:** 10.3389/fimmu.2021.733418

**Published:** 2021-09-17

**Authors:** Qian Zhou, Ran Zhou, Haojun Yang, Huan Yang

**Affiliations:** Department of Neurology, Xiangya Hospital, Central South University, Changsha, China

**Keywords:** myasthenia gravis, immunosuppression, neuromuscular disease, vaccines, infection prophylaxis, autoimmune, COVID-19, SARS-CoV-2

## Abstract

Myasthenia gravis (MG) is an autoimmune disease characterized by muscle weakness and abnormal fatigability due to the antibodies against postsynaptic receptors. Despite the individual discrepancy, patients with MG share common muscle weakness, autoimmune dysfunction, and immunosuppressive treatment, which predispose them to infections that can trigger or exacerbate MG. Vaccination, as a mainstay of prophylaxis, is a major management strategy. However, the past years have seen growth in vaccine hesitancy, owing to safety and efficacy concerns. Ironically, vaccines, serving as an essential and effective means of defense, may induce similar immune cross-reactivity to what they are meant to prevent. Herein, we outline the progress in vaccination, review the current status, and postulate the clinical association among MG, vaccination, and immunosuppression. We also address safety and efficacy concerns of vaccination in MG, in relation to COVID-19. Since only a handful of studies have reported vaccination in individuals with MG, we further review the current clinical studies and guidelines in rheumatic diseases. Overall, our reviews offer a reference to guide future vaccine clinical decision-making and improve the management of MG patients.

## Introduction

Myasthenia gravis, an autoimmune antibody-mediated disease that affects the postsynaptic membrane at the neuromuscular junction, is characterized by muscle weakness and abnormal fatigability (1). Its worldwide prevalence is 150-250 cases per million individuals, with an annual incidence of approximately 8-10 cases per million person-years, although these greatly vary across MG subgroups (2). Despite differences in clinical and pathological manifestations between subgroups, all MG patients share muscle weakness, an autoimmune disease mechanism, and immunosuppressive treatment (IST), which predispose them to infectious disorders (3). Infection, as it had been proved in much research, is closely related to the initial event and exacerbation of MG.

Vaccination is a mainstay of preventing infection in such high-risk populations (4). However, natural infections can result in autoimmune disorders, posing a question as to whether vaccines containing pathogen antigens can also increase the risk of flare or exacerbate autoimmunity in susceptible individuals, in a similar fashion to infection (4). Anti-vaccination lobby groups have described a series of case reports of *de novo* initialization of autoimmune disease or flares of existing autoimmune disease after vaccine administration. Their fears have also been fueled by the insufficient immune responses in protecting MG patients with IST, especially during the pandemic.

These concerns, regarding the safety and efficacy of vaccination, have raised a fervent debate, exacerbated the hesitancy of vaccination, and posed a threat to public health. According to a previous study that investigated poor compliance among MG patients, it is evident that fear of general, non-myasthenic side effects (42.6%), the fear of a myasthenic exacerbation (31.5%), and refusal from their treating physicians (14.8%) are the main reasons for the observed hesitancy (5). A recent survey showed that experienced practitioners are more conservative in vaccine recommendation, despite existing guidelines (6). Little is known regarding the safety and efficacy of vaccination in MG patients. In this review, we will discuss infection- and vaccination-induced effects on the immune system, and address the safety and efficacy of vaccination in patients with myasthenia gravis. We also discuss the status of MG vaccination in relation to COVID-19 vaccines.

## Effect of Infection on MG

Previous studies have shown that infections are closely associated with the onset of MG, and is the largest contributor of MG progression (30%) (3, 7, 8). Initial events triggering MG have been hypothesized inside the thymus, where thymoma accounts for 10% of MG cases and the remaining 90% have unknown causes, among which genetic factors are responsible for less than 50%. Infections act as a major external causal factor since previous reports have associated MG with various viral infections, including Hepatitis B and C, herpes simplex, HIV, West Nile virus, Zika virus, dengue virus, cytomegalovirus, parvovirus B19, human foamy virus, poliovirus, Epstein Barr virus (EBV), and SARS-CoV-2 (9–19). The thymus is a common target organ for infectious diseases that not only alter thymocyte development and export, but also affect the thymic microenvironmental compartment (20). However, MG symptoms are likely to occur long after a triggering infection, which makes it difficult to correlate with a particular infection.

Muscle weakness, IST, and immune dysfunction of MG patients shape the vulnerability to infections which in turn account for MG deterioration. Infections have been identified as the most common cause for precipitating a life-threatening event according to a population-based study from Spain, and associated with 44.3% of emergency department visits and 39.7% of hospitalizations (7, 21). MG exacerbations caused by infections have not been linked to any specific microorganisms.

In attempts to explain the onset or exacerbation of MG after infection, numerous mechanisms have been proposed. Consequently, molecular mimicry, which refers to cross-reactivity between microbial antigens and self-antigens when sharing similarities, has emerged as the most widely accepted mechanism (22). Other mechanisms about autoimmunity after infection include epitope spreading, bystander effect, release of cryptic epitopes, reactivation of memory T cells, activation of superantigens, direct inflammatory damage, formation of immune complexes, expression of MHC antigens on non-immune cells, and patient’s genetic predisposition to autoimmunity (23).

Thus, preventing such infections could protect genetically predisposed groups from developing MG. For MG patients, it is clinically important to avoid serious complications resulting from infections, hence the need for vaccines.

## The Immunology of Vaccines

### The Mechanism of Vaccine

Vaccination, which has rewritten human history since 1796 when Edward Jenner first introduced vaccinia, has become the most cost-effective means for combating infectious diseases. Notable examples of vaccine effectiveness include the eradication of smallpox and the decline in many lethal diseases. Its function depends on the interaction between innate and adaptive responses.

Dendritic cells (DCs), the most efficient antigen-presenting cells (APCs) in the immune system, are the key players in this unique process (24). Specifically, they detect antigens through innate receptors, known as pattern-recognition receptors (PRRs), and present antigens to T cells (24). After inducing a mixture of chemokines and cytokines, DCs transport them to the lymph nodes where activation of T and B cells, as well as production of antibodies, occurs (25). Notably, the specific innate signals received by DCs impact the magnitude and quality of the ensuing T- and B-cell responses, as well as the induction of memory cells (26).

CD4^+^ T cells, activated by antigens that are presented by HLA-class II alleles on APCs, stimulate and sustain B cells’ response. With extreme diversity, HLA molecules are pivotal immune regulatory components encoded by the Major Histocompatibility complex, accounting for individual variation to the immune response against antigens. Those with “favorable” HLA alleles tend to raise optimal and protective antibody titers (27).

**Figure 1:**
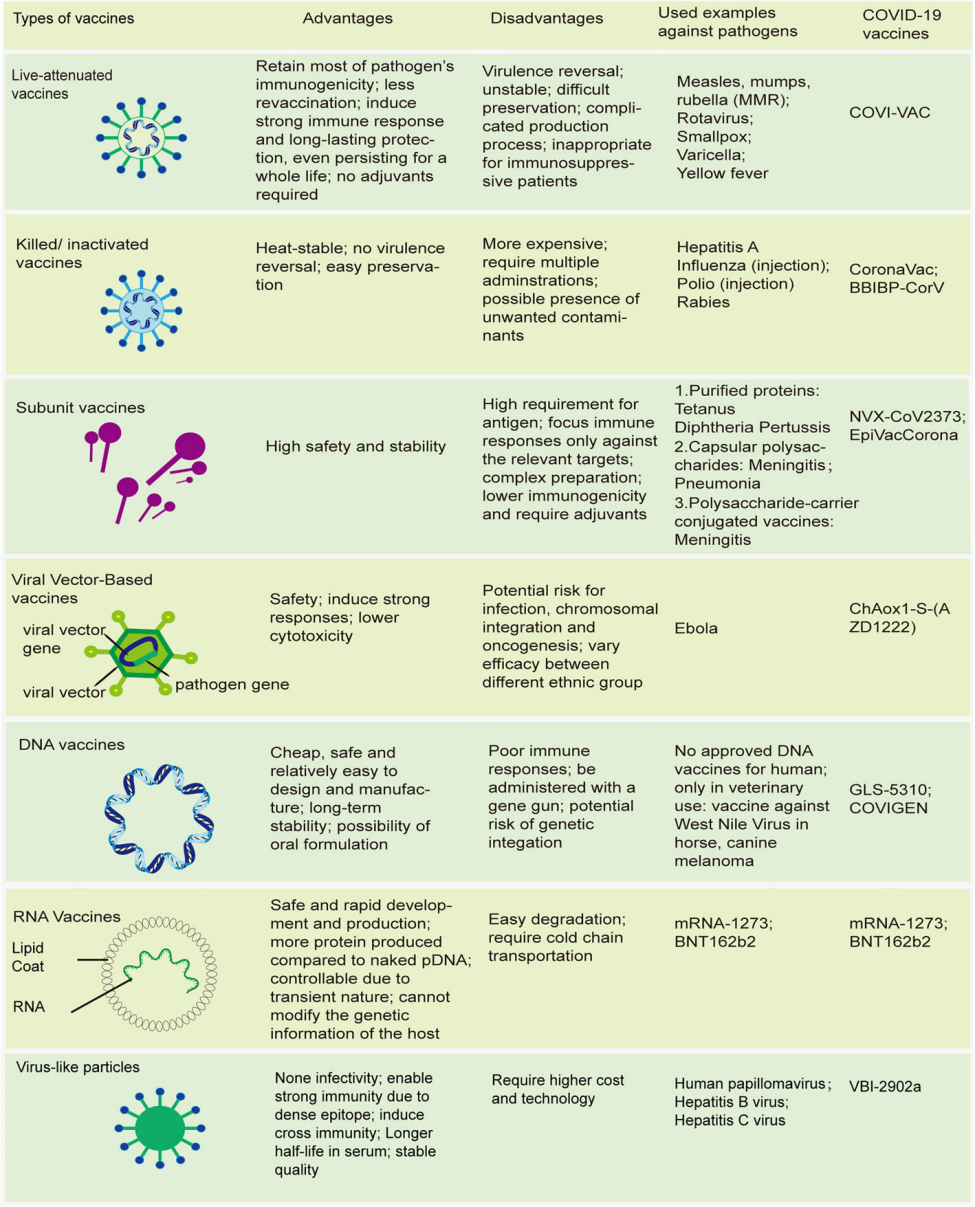
Modern vaccines. Schematics of different types of vaccines; advantages and disadvantages of these vaccines; some traditional vaccines licensed by FDA; novel types of COVID-19 vaccines.

Vaccines have continued to evolve in the epoch of molecular biology and biotechnology, and can now be subdivided into 2 broad groups based on their components (28, 29). The first group comprises live attenuated vaccines, with weakened virulent properties, which simulate the kind of protective immunity induced in people who survive live infection. The others are non-living vaccines (**Figure 1**). Unlike live-attenuated vaccines, which are characterized by high immunogenicity and substantial protection but with a risk of virulence reversal, the second group of vaccines, especially subunit vaccines, exhibit higher safety and fewer side effects, which often impairs their immunogenicity, necessitating the use of adjuvants to improve their potency (30, 31).

Adjuvants, though often not particularly immunogenic by themselves, have been widely used to increase the magnitude and alter the quality of adaptive response, enhance immune responses to vaccine components, and prolong immunological memory. However, their cellular and molecular mechanisms of action remain unclear. Antigens, in combination with adjuvants, enable easier and more effective recognition of “non-self”, enhance the innate immune system and provide a second signal for T cell activation. An ideal adjuvant acts locally at the site of injection to limit system effect, without causing severe side effects (25).

Numerous adjuvants have emerged in the past 100 years, including aluminum salt, virosomes, emulsion adjuvants. Today, many other toll-like receptor (TLR) agonists are still under clinical investigation, with only TLR4 ligand, mono-phosphoryl lipid A (MPLA), licensed (32).

### Vaccines and Autoimmunity

Vaccines, though have saved numerous lives by preventing lethal infection, encountered public inquiry due to evidence of vaccine- and adjuvant-induced autoimmunity using both animal models and human patients (31). As for vaccine-induced autoimmunity, certain pathogenic elements in the vaccine bearing a significant similarity with specific human proteins, can lead to immune crossreactivity and harm similar human proteins, based on molecular mimicry. Molecular mimicry is supposed to facilitate immune tolerance (33–35), but further study found that this tolerance could be attenuated by genetic susceptibility to autoimmunity and environmental factors, especially adjuvants (36). The presence of stimuli including lipopolysaccharides, altered glycosylation patterns, posttranslational modification as citrullination, and chemical modification as oxidation and glycation, can also result in breaking tolerance (37). Furthermore, “Autoimmune/Autoinflammatory Syndrome Induced by Adjuvants” (ASIA syndrome) which refers to a broad spectrum of reactions was proposed by Shoenfeld in 2010, and remains highly controversial entity (38, 39).

Adjuvants improve vaccines’ efficacy through the innate immune system. Generally, pathogens can naturally generate their adjuvant effect through multiple PRRs, although most vaccines need the aid of adjuvants. Most adjuvants are considered immunostimulatory agents and ligands for PRRs, responsible for shaping the innate immune response and producing type-I interferon (IFN-I) which is critical not only in anti-viral mechanism, but also in the pathogenesis of autoimmune inflammatory diseases (AIIDs) (31). Furthermore, signaling *via* TLR3, TLR4, TLR7, TLR8, and TLR9 improves T helper cells type 1 (Th-1) immune responses, while signaling *via* TLR2 (along with TLR1 or TLR6) and TLR5 enhances Th2-type responses (40, 41). These responses have also been shown to influence regulatory T cells (Treg) and Th17 development, which is particularly relevant for AIIDs (42). Although Coffman et al. (31, 43) believed that this innate immune stimulation is short-lived, circumscribed, and does not trigger AIIDs due to insufficient autoreactivity, this hypothesis was refuted with the use of self-antigens or its analogs, and genetic predisposition. For example, the similarity between a peptide sequence of the influenza nucleoprotein A and an extracellular domain of Hypocretin receptor was found to result in narcolepsy after Pandemrix vaccination in the 2009 H1N1 pandemic. Interestingly, HLA-DQB *06:02 allele was found in over 90% of narcolepsy patients, while Canadian vaccinated population without such genetic component did not experience this disorder (36).

### Animal Models

Investigation of viral pathogenesis, safety and efficacy of vaccines, and autoimmune mechanism require animal models which entitle researchers to apply translational research to predict human responses. Non-human primates, a traditional and fundamental model to test new vaccines in clinical trials, turn out to be unreliable, compared with murine which share the highest levels of heptapeptide with pathogens and humans (44). However, rodents, the most widely used models in both preclinical and mechanistic evaluation of vaccines, still have substantial discordance with regards to immune responses with humans. Important distinctions, including the expression and function of TLR, between rodent models and humans, have been found (40). Cross-species differences complicate the interpretation of animal-derived data on humans.

### Mechanism of Vaccination and MG

**Figure 2:**
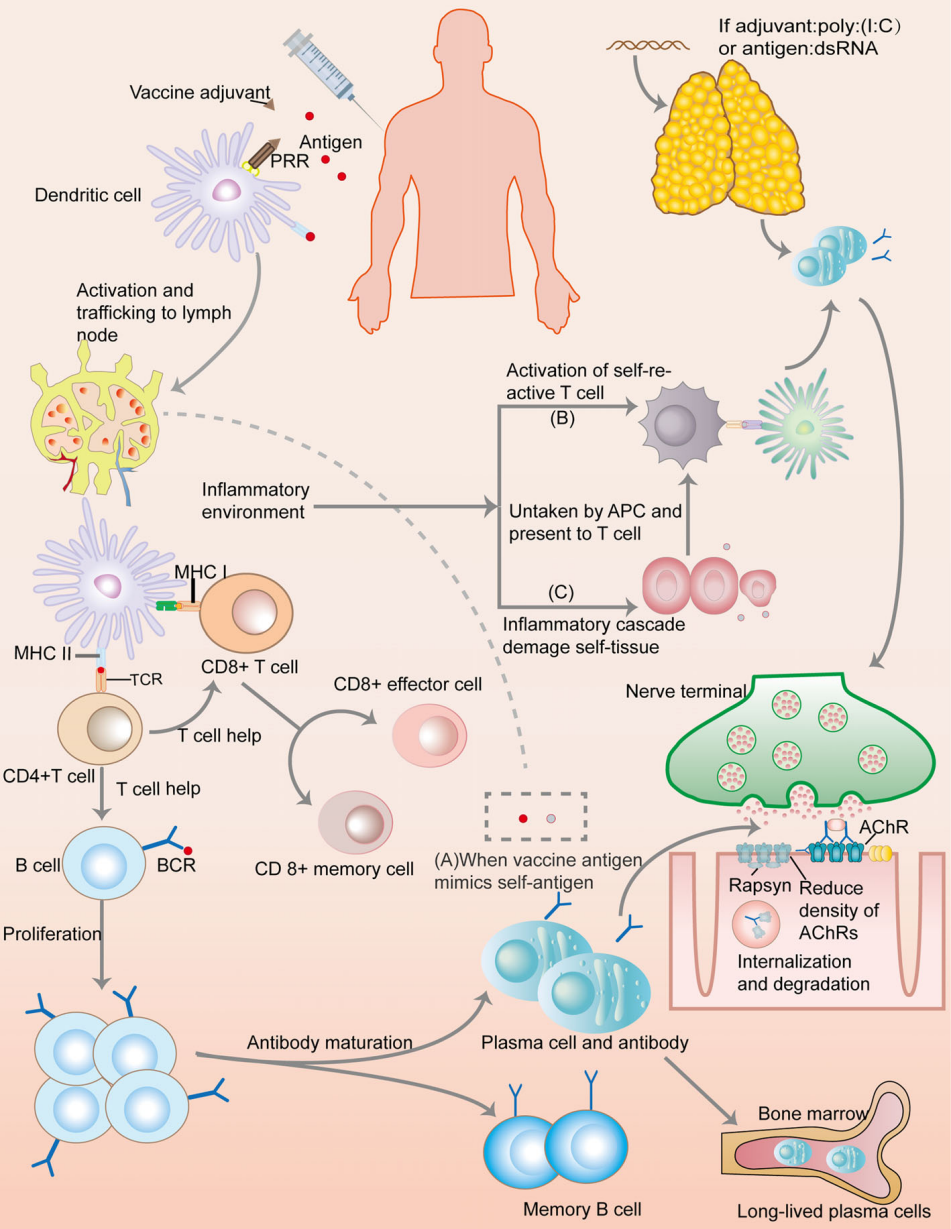
Immunology of a conventional protein vaccine and the possible mechanisms of triggering myasthenia gravis. After a vaccine is injected, antigens would be taken up by dendritic cells (DCs). Combining with adjuvant, pattern recognition receptors (PRRs) deliver danger signal into the DCs, inducing DCs activation and transportation to lymph nodes where the presentation of vaccine antigen by MHC molecules would lead to the activation of T cells through combining to T cell receptor (TCR). With the help of CD4+T cells, B cells undergo activation and proliferation, leading to antibody maturation and the appearance of short-lived and long-lived plasma cells, as well as memory B cells. CD8+T cells also develop into CD8+effector cell and memory cells. This immunization processes create a special inflammatory environment which correlates with the mechanisms of autoimmune: **(A)** Molecular mimicry: If vaccine antigens share great similarity with self-antigens, autoimmune reaction may ensue because the immune response to the vaccine antigens can crossreact with self-antigens. **(B)** Bystander effect: immune responses to vaccine antigens can induce inflammatory signals, inadvertently resulting in the activation of self-reactive T cells, and thus the autoimmune processes. **(C)** Epitope spreading: inflammatory cascades cause tissue damage, and the presentation of self-antigens would trigger the activation of self-reactive T cells. Additionally, if a vaccine contains dsRNA or its analog, such as poly(I:C), it can trigger a thymic overexpression of IFN-β, leading to MG through overexpression of AChR, DCs autosensitization, abnormal recruitment of B cells, and GC formation.

Though numerous autoimmune case reports after vaccination lead to the peaking passion in the exploration of mechanism, little is known regarding the vaccines’ role in triggering or exacerbating MG (**Figure 2**). MG, a prototypic but multifaceted autoimmune disease, involves a spectrum of antibodies (Abs) and a specific mechanism of action. Although the actual underlying mechanisms of MG are unclear, presence of ectopic germinal centers (GCs) and loss of central as well as peripheral tolerance have been discussed (1). High numbers of Th17 cells, follicular Th (Tfh) cells, and dysfunction of Tregs have been shown to promote autoantibody production from B cells and plasma cells (PCs), thereby exacerbating MG pathogenesis (45). Interestingly, the pattern of MG pathogenesis, to some extent, overlaps with the underlying mechanism of vaccine action. For example, Tfh cells are required for GCs, Abs, and long-lived PC responses, not only in MG but also in vaccine immunization (45, 46). Nonetheless, the link between the underlying mechanism of vaccination action and MG remains unclear.

Activation of the TLRs signaling pathways plays a crucial role in triggering and sustaining the inflammatory response and chronicity of MG. Accumulating evidence has indicated that TLRs, mainly TLR2, TLR3, TLR4, TLR7, TLR8, and TLR9 in MG, coupled with aberrant expression or persistent triggering of these receptors, can result in self-sustained inflammation (47–52). For example, a previous study demonstrated that MG thymus with persistent poliovirus infection can synthesize similar quantities of plus and minus RNA strands and VP1 (a viral protein), and express more TLR4, indicating that chronic inflammation and dysregulation of the TLR4 pathways in MG thymus could be attributed to viral persistence (17). Moreover, research has revealed crosstalk between intrathymic EBV with TLR7 and TLR9, a phenomenon that consequently drives massive proinflammatory cytokines, chemokines, and IFN-I produced by thymic epithelial cells (TECs) and plasmacytoid DCs in a genetic susceptibility context (53). INF-I, produced by TLR7 and other TLRs, may form an exacerbating vicious cycle by maintaining TLR7 expression (53). TLR pathways are also involved in vaccine responses, where they control the activation of adaptive immunity. Interestingly, vaccines may contain TLR ligands as adjuvants, or the primary antigens can be TLR ligands themselves, implying that vaccines can trigger and sustain this autoimmune activity. These results suggest that the TLR pathway is an intersection between MG pathogenesis and vaccine action.

MPLA, a licensed TLR4 adjuvant and a derivative of lipopolysaccharide, is far less toxic. Unlike TLR4 in controlling of both the MyD88 and TRIF signaling pathways, MPLA has a bias toward TRIF signaling, and induces high IL-10 expression that could counterbalance the proinflammatory response (40). Currently, there is not report associating MPLA with MG.

However, Polyinosine-polycytidylic acid (poly(I:C)), a TLR3 agonist, can induce thymic changes and trigger MG symptoms by mimicking double strands RNA (dsRNA), a viral nucleic acid associated with viral replication and antiviral mechanism (50). Poly(I:C) can specifically result in the overexpression of acetylcholine receptor α (α-AChR), but not other tissue-specific antigens (TSAs) in TECs. This process is mediated by TLR3, protein kinase R and IFN-β, etc., which are involved in antiviral responses, and observed in MG thymus. Further studies reveal IFN-β plays a central role in thymic events leading to specific overexpression of α-AChRs, TECs apoptosis, and DC autosensitization against AChR. It can cause overexpression of CXCL13 and CCL21 that are associated with GCs development, and induce the B cell-activating factor (BAFF) that favors autoreactive B cell survival (54). Moreover, poly(I:C) are proved to engage in thymic involution by disrupting T-cell development (55, 56). Thus, the injection of vaccines containing specific molecules, no matter from pathogen antigens or adjuvants, which mimics dsRNA and activate TLR3 pathway, can possibly lead to a specific autoimmune reaction against AChR.

## The Safety of Vaccination in MG

### Would Vaccination Trigger MG?

Previous case reports have shown that vaccines can cause MG occurrence, as evidenced by the relationship between MG onset with intravesical Bacillus Calmette-Guerin for bladder cancer and human papillomavirus (HPV) vaccination (57–59). Unfortunately, the mechanism is unclear and requires further investigation. Recent investigation has validated the massive commonality between HPV L1 epitopes and human proteins, i.e., the immune attacks against HPV L1 epitopes may cross-react with human proteins that share the epitopic peptide sequence (37). MG is also reportedly induced and exacerbated by Hepatitis B vaccination, although there is no molecular mimicry between HBsAg and nicotinic AChR (11).

A recent investigation, exploring the molecular and clinical relationship between childhood-onset myasthenia gravis (CMG) and live-attenuated Japanese encephalitis vaccination (LA-JEV), implicated vaccinations in the development of MG, after excluding genetic factors and viral infection (60). Exposing BALB/c mice to a group of Chinese planned immunization programs, researchers found LA-JEV can cause MG-like symptoms in the mice (60). Results later revealed that an 86% identity in a 7-amino-acid-long region was shared by both the human/murine AChR α-subunit (TWTYDGS) and RdRp (TWTYHGS) that is generated during the replication of LA-JEV *in vivo* (60). Based on these findings, it was evident that antibodies against RdRp can cross react with AChR. Notably, the LA-JEV vaccinates nearly 20-30 million children in China every year, but it is in the expanded immunization program in Japan and Taiwan, and not incorporated in the national immunization programs across Europe, North America, and Australia, which might explain the low incidence of CMG in these areas (60). Although this research presents strong evidence owing to the study design that combined retrospective and cohort nature, animal models, epidemiology, and molecular mechanism, the pathology caused by low antibody titer, as well as knowledge of the additional factors associated with immunological profile remains unclear, requiring further clarification.

The analysis of the potential link between vaccines and MG is puzzling. Currently, there exist no general criteria for diagnosing vaccine-related MG, necessitating a case-by-case assessment (61). Consequently, the World Health Organization (WHO) has formulated four basic principles for assessing the adverse events (AEs) of vaccines, namely consistency, strength, specificity, and temporal relation (61, 62). Epidemiology and case reports are valuable clues for deciphering the relationship between vaccines and MG. However, there is a need to consider background incidence, genetic predisposition, environmental factors, and time relevance (clinical data in most clinical trials are collected within 6 weeks but recent research proposes one year after the last vaccination as a maximum theoretical risk interval) (63).

The rate of autoimmune reactions after vaccination is less than 0.01% of all vaccinations worldwide, possibly biased by underreporting. In most countries, reporting such vaccine-related AEs is based on voluntary rather than obligation. Thus, clinicians and patients should be encouraged to report such correlations.

Though for most people, the benefits of immunization trump the potential risks since developing post-vaccination MG is rare, compared with the number of vaccines administered. Then who should be cautious of vaccination? Soriano et al. (64) purposed four groups at risk (1): Persons with prior-vaccination autoimmune phenomena; (2) Persons with a medical history of autoimmunity; (3) Persons with a history of allergic reactions, especially vaccine-related reactions; (4) Persons who are susceptible to autoimmunity, with a family history of autoimmune diseases, presence of autoantibodies, certain genetic profiles, etc.

### Would Vaccination Exacerbate MG?

Influenza vaccines, have been assessed for their safety in MG patients (**Table 1**). In 2009, a population-based study was conducted in Canada, over a 14-year period based on data from healthcare databases. Notably, no association was found between the administration of influenza vaccines and the hospitalization of MG patients (65). However, several factors may have compromised the findings: limited generalizability due to clinicians’ withholding vaccination from younger patients, exclusion of mild worsening of MG, and inability to measure the risk level of influenza vaccination across certain subgroups of MG. A similar conclusion was drawn in an Israel study in 2011, where researchers believed that seasonal influenza and H1N1 vaccines were safe for MG patients (5).

**Table 1:** Summary of epidemiologic studies on immune response in MG vaccinated patients.

Vaccine	Researchers	Study design	Subjects	Safety	Efficacy
Influenza vaccine	ZINMAN *et al.* (65)	Self-matched, case-series method	513 MG patients who were hospitalized within 42 weeks after vaccination; risk interval: first 6w; control interval: final 24w	Relative incidence of hospitalization in the risk interval was 0.84 (95%CI: 0.65-1.09)	–
Influenza and H1N1 vaccines	AURIEL *et al.* (5)	Cohort study; using a questionnaire	74 MG patients (38 patients received influenza vaccine,24 H1N1 vaccine, and 20 both)	(1) In H1N1 vaccine group: the upper 95% corrected CI for neurological side effect is 0.142, and the upper 99% corrected CI is 0.167 (2) In influenza vaccine group: the upper 95% corrected CI is 0.013, and the upper 99% corrected CI is 0.013.	–
Diphtheria and tetanus	Csuka *et al.* (66)	Cohort study	279 SLE; 158 MG; 208 HCs	–	A-DIPHTH levels: HCs and MG (0.14[0.06-0.33] vs 0.10[0.04-0.29], p=0.02) A-TET levels: HCs and MG (1.01[0.37-2.49] vs 1.00[0.38-2.65], p=0.73)
Non-adjuvanted influenza vaccination (Mutagrip^®^)	Tackenberg *et al.* (67)	A double-blind randomized controlled trial	62 MG patients (vaccine group; placebo group)	The difference between groups for AChR-ab-titer change 4.0% [-13.3%, 4.5%] (p=0.28) and for QMG change 0.00 [-0.17%, 0.00] (p=0.79). AE was comparable between groups.	–
Influenza vaccine (Trivalent unadjuvanted inactivated vaccines)	Seok *et al.* (68)	Case-controlled study, using a questionnaire	258 patients (112 men, 185 generalized MG, 133 received an influenza vaccination, and 121 had a common cold or ILI)	MG symptoms aggravated in 10(40%) patients after ILI; only 2 (1.5%) aggravated after influenza vaccination.	–
Tetanus revaccination	Strijbos *et al.* (69)	A prospective, placebo-controlled study	65 MG patients (51 AChR MG, 6 MUSK MG, 9 LEMS); HC group: 20.	Titers of disease-specific autoantibodies remained unchanged after revaccination. Mean increase of QMG score was 1.08 point (P=0.01, 95%: 0.5-1.7) in AChR-MG group. Mean decrease of MG-ADL score was 0.86 point (95%CI: 1.6-0.2)	(1) Compared with HCs, AChR-MG have lower anti-TT IgG (pre, p=0.003, post, p=0.03) All have protective anti-TT IgG titer before TR. (2) IM+ and IM- have lower GMT (IM-, p=0.02; IM+, p<0.01); only IM+ have lower GMT 4w after TR (p<0.01) (3) Increase factor of anti-TT titer after TR were comparable.
Influenza vaccine (contains strains H1N1 pdm09, H3N2 and B/Brisbane/060/08)	Strijbos *et al.* (70)	A prospective, double-blind, randomized, placebo-controlled study	47 AChR MG patients; 47 HCs	No change in AChR-ab titer was observed 4w after influenza vaccination. Total scores of the MGC, QMG, MG-ADL outcome measures were the same before and after vaccination between MG vaccination group and placebo group.	(1) Seroprotective titers (HI>1:40) was achieved in 89.4% MG vs 93.6% HCs for H3N2, 95.7% vs 97.9% for H1N1, and 46.8% vs 51% for the B-strain. Seroprotective titer for these strains was reached 40.4% of MG and in 51% of HCs. (H3N2, p=0.2; H1N1, p=0.7, B-strain, p=0.9) (2) No significant effect on serological response between IM- and IM+ (H3N2, p=0.2; H1N1, p=0.2; and B-strain, p=0.9)

MG, myasthenia gravis; AChR, acetylcholine receptor; MUSK, muscle specific receptor tyrosine kinase; w, week; HC, healthy control; CI, confidence interval; AE, adverse events; ILI, influenza-like illness; LEMS, Lambert-Eaton myasthenic syndrome; A-DIPHTH, diphtheria-antitoxin-IgG; A-TET, tetanus-anti-toxoid-IgG; IM, immunosuppressant; TT, tetanus toxoid; TR, tetanus revaccination; GMT, Geomean titers

In 2017, a Korean study used a recall-based self-report questionnaire to demonstrate that the risk of MG symptom exacerbation following seasonal influenza vaccination was very low (1.5%), while the occurrence of influenza-like illness (ILI) was significantly associated with exacerbation of MG symptoms (40%) (68). Therefore, influenza infection is an apparent risk factor for MG progression, whereas vaccination seems to be an effective way to prevent the progression. However, data in this study did not show whether vaccination conferred protection due to imbalance baseline demography and the poor match between the vaccine strain and the circulating influenza viruses. Unlike the apparent bias presented in the former studies, the Korean study focused on a single influenza season when almost all influenza vaccines used in Korea were trivalent unadjuvanted inactivated vaccines, and it used stratified analysis to minimize biases.

In 2017, the first double-blind, randomized, placebo-controlled trial was reported, providing class 1b evidence that influenza vaccination (Mutagrip^®^) had a positive risk-benefit ratio in MG patients (67). Similar studies later revealed that influenza vaccines (Vaxigrip, RVG 22306) and tetanus revaccination were safe for MG patients, as evidenced by lack of clinical or immunological exacerbation in clinical outcome scores and elevated AChR antibody titers (69, 70).

AChR-ab titer, whose change in the individual is a useful indicator of disease activity, did not see a clinically relevant increase in influenza and tetanus vaccination. Interestingly, one study even observed a pronounced AChR-ab reduction in a subgroup of patients without immunosuppressive therapy, while 3 of 4 examined anti-influenza antibody immunoglobulin classes had a more potent increase, casting doubt on whether the influenza vaccine could inhibit an AChR-ab boost and suppress autoimmune effective B and T cells (67). Additionally, clinical outcome scores, a useful tool to evaluate the disease stability and life quality, did not show an obvious change. QMG scores in one study suggested some worsening of MG with a statistically significant increase of 1 point after 4 weeks, which is far less than the 2 or 3 points accepted as minimal clinically relevant difference, and MG-ADL showed improvement, especially 12 weeks after revaccination (69). However, the use of clinical scores as primary outcome measures have their limitation owing to the large variations in individual levels between pre- and post- revaccination.

Notably, unblinded vaccination of MG patients resulted in more side effects and subjective complaints, relative to blinded subjects given a placebo injection, possibly due to the prejudice among MG patients who considered vaccination to be harmful (69).

These studies did not include patients with severe or unstable MG; hence no meaningful conclusion could be drawn from the groups. In fact, most studies were conducted in the stable and quiescent stage to avoid any misunderstanding associated with safety issues, because some AEs attributed to vaccination actually occur due to underlying disease conditions (71).

Although MG could be theoretically exacerbated by vaccine immunogen contents and adjuvants, results from previous clinical trials indicate that influenza and tetanus vaccines are safe for patients with stable diseases. Since randomized, placebo-controlled trials are the best approaches to assess the potential causal relationship between an AE and vaccination, we suggest that more trials of this kind should be performed to enable accurate evaluation of the safety of other vaccines in MG patients.

## The Efficacy of Vaccination in MG

Although vaccination represents a feasible option for managing a substantial number of infections, some previous studies have indicated that the seroprotection rate is significantly lower in immunocompromised than in immunocompetent patients, casting doubt on the efficacy of vaccination (72, 73). MG, a paradigm of autoimmune disease, requires symptomatic treatment and IST. Studies and guidelines in rheumatic diseases indicate that most live vaccines are contraindicated in patients with IST, while inactivated vaccines generally exhibit a similar safety pattern in immunosuppressed and immunocompetent patients, although the immune response to vaccination can be impaired or even absent with regards to magnitude, breadth, and persistence (74, 75). However, data on the efficacy of vaccines in MG remains scarce.

Efficacy of vaccines refers to suppression of disease incidence among vaccinated subjects, relative to unvaccinated ones, while immunogenicity is the ability of a vaccine to elicit an immune response in a vaccinated individual (76). Research on vaccine efficacy has always been hampered and often used immunogenicity as a proxy, owing to the requirement of complex study designs, large cohorts, and long follow-up periods (77). Besides, the level of protection afforded by vaccination is affected by many factors, including immunosuppressants, age, HLA alleles, disease status, vaccine types, doses, and schedule.

A previous prospective study investigating the humoral immune response to tetanus revaccination in patients with MG or Lambert-Eaton myasthenic syndrome showed that IST slightly reduced pre- and post-tetanus antibody titers, but a significant humoral response could still be evoked with a median 6-fold increase factor of the tetanus antibody titer (69). These findings corroborated those from a prior study which found no significant differences in antibody responses and immune protection against diphtheria and tetanus in patients with systemic lupus erythematosus (SLE), and MG, relative to healthy subjects (66). Another study evaluating the efficacy and safety of influenza vaccines in MG patients in 2019 reported that a post-vaccination seroprotective titer for three strains of seasonal influenza vaccine was reached in 40.4% and 51% of AChR MG subjects and healthy controls, respectively (70). In these studies, IST comprised prednisolone, azathioprine, mycophenolic acid, and cyclosporine, as well as a combination of IST. Although IS therapy or thymectomy status did not significantly influence post-vaccination geomean titers, these studies did not measure the specific effects of a single drug, due to the small size of treatment subgroups and frequent combination of IST.

Because of the limited data on the efficacy of vaccines in MG, we analyzed results from studies in other AIIDs and concluded whether IST diminishes the vaccine response depends on the immunosuppressive mechanism and the immune profile of vaccines. AIIDs patients with IST tend to exhibit both similar and lower responses to vaccines compared with healthy individuals. It is ironic that those who most need protection due to their increased infection risk, least benefit from vaccination (78). Our findings indicate corticosteroids has a contradictory effect on the development of protective antibodies after vaccination (79, 80), while rituximab (RTX), a B-lymphocyte-depleting monoclonal antibody, severely inhibits immune response, which is partially restored 6-10 months after RTX administration (72, 73).

Pneumococcal polysaccharide vaccine (PPSV) consists almost exclusively of capsular polysaccharides, which provokes a minimal T cell-mediated antibody production in B cells, while Pneumococcal conjugate vaccine (PCV), conjugating to the diphtheria toxoid CRM197, elicits a robust T cell-dependent immune response (77). Thus, immune responses should be more profound after PCV than after PPSV. However, the effect is exactly contrariwise in patients with IST (77). It is hypothesized that IST mainly compromises cellular immunity, reducing the response rate more severely in PCV than PPSV (77). However, abatacept (ABA), a selective T-cell co-stimulation modulator, can impair PPSV serologic responses (81). This can be explained by the inhibition of B-cell differentiation which requires T-cell help, and polysaccharide antigens cannot be considered completely T-cell independent because they are enhanced by T-cell help.

A latest meta-analysis proved AIID patients with IST presented impaired serologic response, compared to healthy individuals and patients without IST. Patients using anti-tumor necrosis factor α (TNFα) maintained a more favorable immune response to pneumococcal vaccination, compared with those with other IST (77). Likewise, Hua and Kapetanovic reported PPV-23 responses were normal in anti-TNFα, but reduced in Methotrexate (MTX) (82, 83). Disease-modifying anti-rheumatic drugs (DMARDs) exert their effect by blocking of clonal expansion of effector T- and B-cells, while anti-TNFα specifically interferes with the immune system by reducing migration of dendritic cells, inhibiting T cell activation, and reducing memory cell survival (77). This may account for the less negative effect on the immune response with anti-TNFα than with DMARDs. IL-6 also plays a vital role in B cells differentiating into plasma cells. Tocilizumab (TCZ), an IL-6 inhibitor that should hamper antibodies, did not impact response compared with healthy controls (HCs) (84). Thus, some researchers deduce the immune response to vaccination is not influenced in the same way by biologic agents. Treatment target on B cells (RTX) or T cells (ABA) may exert deleterious impact, whereas anti-cytokine therapies (TCZ and anti-TNFα) could maintain humoral responses to vaccines (85).

However, these results, mainly from rheumatoid arthritis, contradict in Crohn’s disease, in which anti-TNFα also severely lowers the antibody titer after pneumococcal vaccination, posing a question of whether results attained from other diseases can apply in MG (86).

Patients exposed to different ISTs are expected to exhibit varied responses to different vaccines, due to the different underlying mechanisms of immunosuppressive drugs and variable immunological patterns of vaccines. Efficacy of vaccination in MG patients with different ISTs requires further clarification, since results obtained from other AIIDs do not directly apply to it. High-quality clinical trials are required to affirm vaccine efficacy in MG patients treated with different IST.

Age of vaccination is another factor that affects vaccine efficacy (87). For example, previous studies have shown that it is somewhat difficult to induce strong immune responses in the first year of life or in older periods, particularly among individuals older than 75 years (66, 68). Previous evidence has also indicated that growing older compromises protection after vaccination (66, 68). Moreover, vaccine doses and administrative schedules also impact protection, since immunocompromised patients often require larger doses or frequent administration (77). Thus, better vaccine-mediated protection can be obtained before the initiation of immunosuppressive treatment.

## Risk-Benefit Assessment of Vaccination in MG

Though previously mentioned research on vaccination in MG patients is scarce and mainly about influenza vaccines, nearly all the evidence supports vaccine-related worsening of MG is rare (5, 65, 67, 68, 70). The antibody response in MG is not different from that in healthy subjects, even in those with IST except RTX (70). Study evaluating the cause of death in Swedish MG patients reveals influenza/pneumonia is a striking contributor (88). A 10-year longitudinal study found a significant 48% reduction in mortality and a 27% reduction in hospital admissions after influenza vaccination in AIID patients (89). Thus, most MG specialists believe the benefits of vaccination outweigh any small risks in possible transient MG symptoms exacerbation (90). Guidelines also recommend influenza and pneumococcal vaccine for AIID patients, which preferably be administered during quiescent disease (91). Patients before Eculizumab treatment should consider be immunized with meningococcal vaccines (92). Discussion with doctors when considering a vaccine is necessary to assess the risk-benefit of vaccination (90, 91).

## COVID-19 Vaccines in MG Patients

### SARS-CoV-2 Infection and MG

COVID-19 was declared as a pandemic by WHO on March 11th, 2020. To date, accumulating evidence has confirmed that SARS-CoV-2 is linked to MG. 10 case reports of new-onset MG following COVID-19 have been analyzed with the following features: mean age 51 years, male gender (6), time interval between COVID-19 and MG (5-56 days), generalized (7), bulbar and/or ocular symptoms (5), anti-AChR antibodies (9) and anti-muscle specific kinase antibodies (anti-MUSK) (2) (93, 94).

Many plausible mechanisms have arisen to explain such causal relations. First, COVID-19 may be a disease of the nicotinic cholinergic system (95). Sequences on the SARS-CoV-2 proteins, similar to neurotoxin, can bind to AChRs and block the function of acetylcholine. Second, SARS-CoV-2 peptide CFLGYFCTCYFGLF aligns neuronal acetylcholine receptor subunit alpha-2 with 7 residue matches, affirming that antibodies against SARS-CoV-2 can crossreact with the human tissue, thereby causing autoimmunity (96). However, these studies did not analyze conformational or non-linear epitope (97, 98). Third, researchers have also speculated that SARS-CoV-2 can unmask a previously non-symptomatic MG or COVID-19 patients incidentally suffer an overlapping occurrence of MG (99). Furthermore, SARS-CoV-2 produces a proinflammatory milieu and cytokine storm, resulting in immune dysregulation and disrupted self-tolerance. IL-6, which is associated with a higher mortality rate in COVID-19 patients, also correlates with MG progression (100). Lastly, drugs like hydroxychloroquine sulfate and azithromycin during treatment can also trigger MG.

Moreover, mounting case reports show COVID-19 links to MG progression. Viral infection is perceived to trigger autoimmunity through augmentation of T cell signaling, thereby contributing to the proinflammatory environment, triggering hyper-reactive antiviral immune responses, epitope spreading, and effects of fever on the neuromuscular junction (3, 101).

The point is of interest since often, the autoimmunity after infection somewhat corresponds to the autoimmunity after vaccination. The aforementioned occurrence and deterioration of MG after COVID-19 infection implies that the protective antiviral antibody immune response can become a pathogenic attack against the human organism, while the adjuvanted anti-SARS-CoV-2 vaccines with higher immunogenicity can also elicit autoimmune response compared to SARS-CoV-2 infection.

### SARS-CoV-2 Vaccines Hesitation

Vaccines, serving as a cornerstone in mastering the COVID-19 pandemic and achieving herd immunity, is rapidly evolving. Unlike traditional vaccines whose development pipeline span several years, sometimes even 15 before approval and distribution, SARS-CoV-2 vaccines development has been revolutionized, with 18 vaccines in Phase III clinical trial and 5 ending Phase III with positive results within 2 years (102). Consequently, such rapid speed to attain an insufficiently vetted vaccine increases the risk of trading freedom from COVID-19 to an autoimmune assault (97). Unfortunately, the risk of cross-reactivity increases, since the current preclinical tests cannot measure it (44, 103). Although interim Phase III safety data from some vaccine trials have been largely positive, these studies failed to measure rare adverse effects due to a limited number of participants coupled with a lack of long-term efficacy because of trial results obtained from the last few months. It could be hard to identify some AEs of such vaccines produced utilizing new technologies. Moreover, Phase III trials currently underway mainly targetted on a healthy population.

An online survey on people’s acceptance of COVID-19 vaccines showed that 54.9% of patients with rheumatic and musculoskeletal diseases (RMDs) were willing to get vaccinated, compared to the rate of HCs, 82.3%. Their refusal was mostly disease-linked, and reasons included fear of AEs and disease worsening (104).

### Safety of SARS-CoV-2 Vaccines in MG

To date, several types of COVID-19 vaccines have undergone clinical trials and some have been approved for emergency use. Among them, RNA-based vaccines, including Pfizer/BioNTech (BNT162b2) and Moderna (mRNA-1273) mRNA, which were the earliest to be approved, serve as both immunogen and adjuvant, and have generated contrasting effects on innate immune responses (105, 106). Specifically, they are considered safe, without infectivity and integrating vector. With possible modification, they can be effective by increasing mRNA translation, and maintain the production of IFN-I and proinflammatory cytokines, which are desirable in countering the virus (106). On the other hand, the adjuvanticity of these vaccines is based on TLR 3,7,8,9 agonists, distinct from previous vaccines, and is a common pathogenic mechanism in autoimmunity (107). Researchers believe that some modifications, including the incorporation of modified nucleosides, can increase vaccine efficacy but reduce innate immune activation (106).

Following the strict guidelines from WHO to assess the causality of adverse events following immunization after COVID-19, 2 new-onset MG cases were identified after the second dose of BNT162b2 vaccine, one being severe (107). A case of MG crisis after the second dose of COVID-19 vaccine has also been reported (108). The new-onset and flare of MG all occurred within 1 week after the second dose. Noteworthily, before vaccination, this MG patient with a 5-year course still maintained on prednisone 7.5-milligram tablet daily and pyridostigmine 60-milligram tablet six times daily, which meant he didn’t have MG remission. Though steroids can reduce cytokine expression, lymphocyte differentiation, and proliferation, it is possible that this patient was not medically optimized and still developed a cytokine storm (108).

There is few research hitherto about MG course after COVID-19 vaccination. Several studies investigating the safety of SARS-CoV-2 mRNA vaccines in patients with stable RMDs revealed minor side effects and no distinct impact on RMD activity (109, 110). A recent multicenter study reported no evidence of significant disease flares, but the occurrence of herpes zoster after BNT162b2 mRNA vaccination in subjects with low and even no IST is worth investigating (80).

### Efficacy of SARS-CoV-2 Vaccines in MG

Research investigating the efficacy of SAR-CoV-2 vaccines in MG patients is scarce. Extrapolating from studies in RMD, rituximab, a B-cell depleting agent, is significantly associated with a higher seronegative rate (80, 111, 112). Thus, most guidelines suggest patients on RTX should be vaccinated either one month before initiation of the therapeutic scheme or 6-8 months after the RTX infusion (113). However, previous reports showed some patients with RMD on RTX therapy still developed adequate titers of antibodies against SARS-CoV-2 despite having undetectable B cells (114, 115). B cells are suspected to play a comparatively lesser role in the clearance of virions. Preclinical and early results from human trials showed that human after mRNA vaccination had Th-1 skewed T cell immune responses with RBD-specific CD8+ and CD4+ T cells. Those on B cell-depleted treatment with a deficient humoral response may still be protected by the cellular response (116). Moreover, those who had adequate antibodies used lower doses of RTX or just initiated this therapy (115).

Patients on the scheme of mycophenolate mofetil or abatacept exhibited significantly lower SARS-CoV-2 antibody titers (80, 111, 112). The blunted response was also associated with glucocorticoids, even if its doses were low (6.2mg/day), which precluded the consideration of dose-dependent effect (80). MTX, as monotherapy or in combination with other drugs except abatacept or rituximab, elicited a slightly reduced response (80). Older age is another factor contributing to the reduced immunogenicity (80).

From the standpoint of particular autoimmunity, rheumatoid arthritis, antineutrophil cytoplasmic antibody-associated vasculitis, and idiopathic inflammatory myositis are at risk of blunted response to the vaccine compared with other diseases (80). Further studies are needed to determine the factors, including drugs, doses, duration, timing of IST that influence the antibody responses to COVID-19 in MG patients.

### Risk-Benefit Assessment and Should MG Patients Be Vaccinated?

Today, MG patients with COVID-19 infections experience a highly variable course and outcome. The percentage of patients who need hospitalization and/or experienced MG exacerbation varies across studies (117). MG patients might face a higher risk of severe outcome, especially those with respiratory muscle weakness, older age, other and other medical or neurologic diseases (118). Data from a global, physician-reported registry of 91 MG with COVID-19 infection presented 63(69%) demand for hospitalization, 36 (40%) requiring rescue therapy (e.g., IVIG, PE, or steroids) due to disease worsening or crisis of MG, 39 (43%) complete recovery or discharging home, and 22 (24%) death (119). These figures are valuable because of analyzing a number of patients, but they did not consider international variations in the infection course. Unexpectedly, studies in the French and Polish cohorts showed COVID-19 had limited effect on most MG patients, while high myasthenia gravis foundation of America (MGFA) class (≥IV) before COVID-19 was associated with severe COVID-19 (117, 120). IST did not have a significant impact on infection risk (120, 121). Another important aspect influencing the infection course in MG patients is international differences in the number of vaccinated populations as those who get vaccinated will form a “cocoon effect” to protect those who cannot be vaccinated (117).

Owing to a lack of clinical vaccine trials in patients in MG and the inconsistency between RMD and MG, there is no direct evidence about COVID-19 vaccine safety and efficacy in MG patients. Recently, MGFA supports the potential benefits of SARS-CoV-2 mRNA vaccines outweigh the risks, and recommend patients with IST to discuss getting an additional mRNA vaccine dose with their treating providers (122). For healthy subjects without the conditions mentioned by Soriano et al. (64), it is unlikely to develop new-onset MG after vaccination. For MG patients who must be vaccinated, they had better reach minimal manifestation stage or at least disease quiescent stage.

To ensure the uttermost safety and best efficacy, patients should consult their clinicians to discuss the following: their attitudes, intent, safety and efficacy concerns about vaccination, local incidence of COVID-19, individual circumstances (e.g., disease activity, medications, comorbidities) (123). Risk and benefit assessment should be carried out on a case-by-case basis. The usual post-vaccination observation time that lasts 15-30 minutes does not fully capture the AEs that may appear 1-2 weeks after vaccination (108). Both patients and clinicians should carefully monitor AEs since those who had previously been COVID-19 infected might experience AEs after first doses, and who had not been infected could get AEs after the second dose (124).

### Which Vaccine Is Preferable in MG Patients?

All vaccines target at least on the epitopes of S protein or its RBD sequence that either vectored or presented in different ways (125). Right now, with a lack of evidence, there is no preference for one COVID-19 vaccine over another. Firstly, it will take a long time before industrial policies and national political issues allow such comparative assessment of these vaccines and set international standards. Secondly, geographic contexts, economic conditions, supply, storage, and schedule of administration are also necessary elements to determine the choice. The evaluation will continue. Future cohorts to monitor vaccine effectiveness and safety among individuals with rare conditions will guide preferred orientation according to age, immune and medical status, etc.

## Discussion and Perspectives

The relationship between humans and vaccines is no doubt intricate and complex, with new layers still unraveling. Although numerous vaccines have been developed, totally transforming human life, their underlying mechanism of action, as well as the association with myasthenia gravis remain unknown, necessitating further studies. Theoretically, vaccines could trigger and enhance the progression of MG, in a similar fashion to infection, and immunosuppressive treatment may impair immune response, but recent experimental results have demonstrated the safety and efficacy of influenza and tetanus vaccines. Molecular mimicry is a commonplace topic, a validated reality rather than a fantasy. Thus, to select peptide epitopes for safe vaccine design, analyzing already proven immunoreactive epitopes and filtering out the peptide with cross-reactive potential becomes necessary.

Since the direct evidence about COVID-19 vaccines in MG patients is limited, more data is required to validate their safety and efficacy. Future studies should not only focus on the molecular aspects, but also aim at providing more clinical evidence to enable MG patients and their physicians to make decisions.

## Author Contributions

HY developed the idea, participated in the study design and critical revision of the manuscript. QZ did the literature research, framed the outline, and wrote the manuscript. RZ gave helpful advice, and critically revised the draft. HJY gave helpful advice. All authors contributed to the article and approved the submitted version.

## Conflict of Interest

The authors declare that the research was conducted in the absence of any commercial or financial relationships that could be construed as a potential conflict of interest.

## Publisher’s Note

All claims expressed in this article are solely those of the authors and do not necessarily represent those of their affiliated organizations, or those of the publisher, the editors and the reviewers. Any product that may be evaluated in this article, or claim that may be made by its manufacturer, is not guaranteed or endorsed by the publisher.

## Abbreviations

MG, Myasthenia gravis; IST, immunosuppressive treatment; EBV, Epstein Barr virus; PRRs, pattern-recognition receptors; APCs, antigen-presenting cells; DCs, Dendritic cells; TLR, toll-like receptor; AChR, acetylcholine receptor; MPLA, mono-phosphoryl lipid A; IFN-I, type-I interferon; AIIDs, autoimmune inflammatory diseases; Treg, regulatory T cells; Tfh, follicular Th; Th1, T helper type 1; PC, plasma cell; GCs, germinal centers; TECs, thymic epithelial cells; HPV, human papillomavirus; MUSK, muscle specific receptor tyrosine kinase; poly(I:C), polyinosinic-polycytidylic acid; ASIA, autoimmune/inflammatory syndrome induced by adjuvants; Abs, antibodies; dsRNA, double strands RNA; TSAs, tissue-specific antigens; CMG, childhood-onset myasthenia gravis; LA-JEV, live-attenuated Japanese encephalitis vaccination; SLE, systemic lupus erythematosus; WHO, World Health Organization; ILI, influenza-like illness; CS, corticosteroids; RTX, rituximab; PCV, Pneumococcal conjugate vaccine; PPSV, polysaccharide vaccine; ABA, abatacept; DMARDs, Disease-modifying anti-rheumatic drugs; TNFα, Tumor necrosis factor α; TCZ, Tocilizumab; RMDs, rheumatic and musculoskeletal diseases; HCs, healthy controls; AEs, adverse events.
